# Angiotensin II receptor expression and relation to *Helicobacter pylori*-infection in the stomach of the Mongolian gerbil

**DOI:** 10.1186/1471-230X-10-3

**Published:** 2010-01-14

**Authors:** Peter Hallersund, Herbert F Helander, Anna Casselbrant, Anders Edebo, Lars Fändriks, Anders Elfvin

**Affiliations:** 1 Department of Gastrosurgical Research and Education, Institute of Clinical Sciences, Sahlgrenska Academy at University of Gothenburg, Gothenburg, Sweden

## Abstract

**Background:**

The role of the renin-angiotensin system in gastric physiology and disease has as yet been sparsely explored. The first aim of the study was to investigate the baseline presence and location of angiotensin II receptors (AT1R and AT2R) in the stomach of the Mongolian gerbil. A second aim was to elucidate whether the presence of *H. pylori *infection is associated with changes in the expression of these receptors.

**Methods:**

*H. pylori*-negative and *H. pylori-*infected (strain SS1 or TN2GF4) male Mongolian gerbils were investigated. The stomachs were examined at six or 12 months after inoculation by the use of immunohistochemistry, western blot and microscopic morphometry.

**Results:**

AT1R and AT2R were located in a variety of cells in the gerbil gastric wall, including a subpopulation of endocrine cells in the antral mucosa and inflammatory cells infiltrating *H. pylori*-infected stomachs. Gerbils infected with the SS1 strain showed a significantly increased antral AT1R protein expression and an increased number of infiltrating polymorphonuclear leucocytes (PMNs) at 12 months. The AT1R protein expression correlated with the number of PMNs and the antral expression of myeloperoxidase.

**Conclusions:**

Angiotensin II receptors are present in a variety of cells in the gastric wall of the Mongolian gerbil. The results indicate an influence dependent on the *H. pylori *strain on the gastric AT1R expression and a relationship between gastric AT1R expression and mucosal PMNs infiltration.

## Background

The role of the renin-angiotensin system (RAS) in gastric physiology and disease has as yet been sparsely explored. Some studies have reported actions of angiotensin II (Ang II) on mucosal blood flow, acid secretion and smooth muscle contraction [1-3]. Others have implicated the influence of RAS in stress induced gastric injury and ischemia/reperfusion damage of the gastric mucosa [4,5].

The classical endocrine character of RAS is well known for its effects on hemodynamic regulation and body fluid homeostasis. Less is known about the regulatory impact of tissue based local RAS that has been demonstrated in a number of organs, e.g. the brain, pancreas, esophagus and colon [6-8]. Interstitial production of Ang II may occur following local production of angiotensinogen (AGT), ACE and renin, or through alternative pathways including cleavage of circulating AGT by other locally expressed enzymes such as cathepsin D and chymase [9]. Ang II works principally through two receptors, designated Ang II type 1 receptor (AT1R) and Ang II type 2 receptor (AT2R). Consequently, mapping the expression and location of Ang II receptors is of great importance in exploring potential Ang II signalling in different physiological and pathological settings. In recent years RAS has been shown to be involved in various pathological conditions such as inflammation, wound healing and carcinogenesis [10,11]. Epidemiological studies have also demonstrated associations between RAS related gene polymorphism (SNPs) and the development of peptic ulcer and gastric cancer [12,13]. However, associations between tissue expression of Ang II receptors and *Helicobacter pylori *infection have not been reported. The potential of RAS to modulate local inflammatory reactions makes it of interest to investigate its relation to the well-defined gastritis evoked by *H. pylori*.

In the present study, we examined the expression of Ang II receptors in the uninfected and *H. pylori*-infected Mongolian gerbil. This animal model is interesting because it can easily be infected with *H. pylori*, giving a chronic active inflammation with pathological changes that mimic most of the lesions found in humans, including gastric ulcers, gastric intestinal metaplasia and a gastric cancer-like picture [14]. Whether *H. pylori *infection alone causes gastric cancer in Mongolian gerbils remains under debate [15].

A first aim of the study was to investigate the baseline presence and location of the AT1R and the AT2R in the stomach of the Mongolian gerbil. A second aim was to elucidate whether the presence of *H. pylori *infection is associated with changes in the expression of these receptors.

## Methods

### Animals

The experiments were approved by the Ethics Committee of Experiments on Animals, University of Gothenburg. Thirty specific pathogen free (SPF) male Mongolian gerbils (Charles River, Uppsala, Sweden), seven weeks of age, were used. They were randomly separated into three groups of equal size, consisting of one control group and two groups that were to be infected with *H. pylori*. Five animals were kept in each cage and the room was regulated with respect to temperature (18-22°C), humidity (about 55%) and light/dark cycle (12/12h). The gerbils had free access to food (2016F, Harlan Tek. Lab, Blackthorn, England) and drinking water. They were allowed one week of acclimatization before the inoculation.

### Bacterial strains and inoculation

Two different *H. pylori *strains were used for inoculation, the TN2GF4 strain [16] and the Sydney strain 1 (SS1) [17]. Both strains are known to cause a chronic active inflammation in the gerbil gastric antral and corporal mucosa. The bacteria were grown on Columbia plates. These cultures were then used to inoculate 500 ml Brucella broth containing 5% newborn calf serum, 10 μg/ml vancomycin and 5 μg/ml trimetoprim. The flasks were incubated for 24 hours under micro aerobic conditions at 37°C. The bacteria were examined by phase contrast microscopy before being administered to the animals. The gerbils were infected with 0.5 ml of the bacterial suspension or Brucella broth only (controls) using a feeding needle. Viable counts were made in the suspension to determine the actual infectious doses, which were 7 × 10^7 ^units and 4 × 10^7 ^units for SS1 and TN2GF4, respectively.

### Time course and sacrifice

Following a 24-hr fasting period with free access to drinking water, five animals from each group were sacrificed six or 12 months after inoculation. The animals were anesthetized by intraperitoneal injection of medetomodin (0.5 mg/kg) and ketamin (75 mg/kg). A midline laparotomy was performed and the stomachs were then removed and opened along the major curvature. One half was used for culture, and specimens were collected from the other half for histological analyses and western blot analysis. The animals were sacrificed by cardiac incision. Histopathological findings and bacteriological status have been reported previously [14].

### Immunohistochemistry

The full wall specimens (antrum and corpus) were collected immediately after the opening of the stomachs, fixed in Histofix (Histolab products AB, Gothenburg, Sweden) at room temperature (RT) overnight and then rinsed in PBS for 24 hours. Specimens were subsequently dehydrated, embedded in paraffin, cut into 5-*μ *thick sections and mounted on glass slides. Before immunohistocemical staining, sections were deparaffinized and boiled in citric acid buffer (0.01 M, pH 6.0, 10 min). The Immunocruz TM Staining System (Santa Cruz Biotechnology, Santa Cruz, CA, USA) was used for the immunohistochemistry protocol. After inhibition of endogenous peroxidase activity, non specific binding of secondary antibodies was blocked by incubation with normal goat serum for 30 min at RT. The following two polyclonal primary antibodies, dilutions and incubation times were then used: rabbit anti-AT1R (N-10, Santa Cruz Biotech, 1:100 in PBS, 2 hr at RT) and rabbit anti-AT2R (H-143, Santa Cruz Biotech, 1:100 in PBS, 2 hr at RT). After incubation with primary antibodies, sections were washed three times in PBS and probed with a biotinylated secondary antibody; the complex was detected using horseradish peroxidase (HRP)-streptavidin and the color was developed using 3,3'-diaminobenzidine (DAB).

An unexpected, strong staining of cells with a typical appearance of endocrine cells was noted after immunohistochemical staining with this peroxidase based method. Immunoflourescens labelling was done to further rule out the possibility that this staining was a result of endogenous biotin, of endogenous peroxidase activity or of unspecific binding of the secondary antibody. The following protocol was used: after boiling in citric acid buffer, non specific binding of secondary antibodies was blocked by incubation with normal goat serum (sc-2043, Santa Cruz Biotech) for 30 min at RT. Sections were incubated with primary antibodies as above. The slides were then washed three times in PBS and probed with a secondary antibody of Alexa Flour 488 conjugated goat anti-rabbit IgG (A-11034, Molecular Probes Inc, Eugene, OR, USA), diluted 1:400 in PBS for 1 hr at RT. The slides were next washed three times in PBS and mounted with fluorescence mounting medium (DakoCytomation, Glostrup, Denmark), after which images were captured with a fluorescence microscope. To confirm the location of AT1R in endocrine cells, double immunostaining was performed using the above described peroxidase based protocol for AT1R and the above described immunoflorescens labelling protocol for a primary antibody directed against Chromogranin A (C-20, Santa Cruz Biotech, 1:100 in PBS, 2 hr at RT). Non specific binding of the secondary antibody (Alexa Flour 568-conjugated donkey anti-goat IgG, A-11057, Molecular Probes Inc, 1:800 in PBS, 1 hr at RT) was blocked by incubation with normal donkey serum (sc-2044, Santa Cruz Biotech). Preabsorpion with an excess (5×) blocking peptide (sc-1173P, Santa Cruz Biotech) was performed as a control for the specificity of the AT1R antibody, while the primary antibody was omitted for the AT2R.

The gerbil AT1R and AT2R have >90% amino acid homology sequence identity with human, rat and mouse Ang II receptors [18,19]. In gerbils, as in rats and mice, there are two AT1R subtypes (AT1aR and AT1bR) that are encoded by different genes but with 95% amino acid sequence homology. These receptor subtypes are known to be differentially localized and regulated but have a similar affinity for Ang II. Because of high amino acid sequence homology between the gerbil AT1aR and AT1bR, we assumed equal binding affinity to the AT1R subtypes for the AT1R antibody used in the present study. Moreover, the antibodies used for staining Ang II receptors are established by the manufacturer in mice, rats and humans. Therefore, to confirm the specificity of these antibodies in gerbils, sections from paraffin embedded blocks of normal gerbil and human adrenal glands were stained as above (except that both primary antibodies were diluted 1:50 in PBS) and the distribution patterns for the Ang II receptor antibodies were studied. The adrenal distribution studies showed that the anti-AT1R antibody predominantly stained adrenal cortical cells in the zona glomerulosa (staining of the capsule surrounding the gland, vascular smooth muscle cells (VSMCs) and endothelial cells was also noted) in both gerbil and human sections. The anti-AT2R antibody stained adrenal medulla cells and cells in the zona glomerulosa in gerbil and human adrenal gland (staining of VSMCs and endothelial cells was also noted using this antibody). These results agree with previously reported studies [20], and the strong similarity of staining patterns in the gerbil and the human adrenal gland support a general usefulness of the antibodies in gerbil tissues.

### Western blot

After opening the stomachs, full thickness antral wall specimens were immediately collected, frozen in liquid nitrogen and stored at -70°C. The specimens were homogenized on ice (Polytron, PT-MR 2100, Kinematica, Switzerland) in buffer A (10% glycerol, 20 mM Tris-HCL pH 7.3, 100 mM NaCl, 2 mM phenylmethylsulfonyl fluoride, 2 mM EDTA, 2 nM EGTA, 10 mM sodium orthovanadate, 10 μg/ml leupeptin and 10 μg/ml aprotinin) [21] and centrifuged at 30,000 g for 30 min at 4°C. The pellets were resuspended in buffer B (1% NP-40 [Sigma-Aldrich, Stockholm, Sweden] in buffer A) and stirred at 4°C for 1 hr before centrifugation at 30,000 g for 30 min at 4°C. The supernatants were analyzed for protein content by the method of Bradford [21] and stored at -70°C until further analysis. Samples were diluted in SDS buffer and heated at 70°C for 10 min before being loaded on a NuPage 10% BisTris gel (Invitrogen, Carlsbad, CA, USA). One lane was loaded with prestained molecular weight standards (SeeBlue™, Invitrogen AB, Lidingo, Sweden), and whole cell lysates of PC-12 (for AT1R; sc-2250, Santa Cruz Biotech), HEP G2 (for AT2R; sc-2227, Santa Cruz Biotech) and HL60 (for myeloperoxidase (MPO); 12-354, Upstate Lake Placid, NY, USA) were used as positive controls. Polyvinyldifluoride membranes (Amersham, Buckinghamshire, UK) were incubated with polyclonal rabbit antibodies directed against AT1R (N-10, Santa Cruz Biotech), AT2R (H-143, Santa Cruz Biotech) or MPO (07-496, Upstate). An alkaline phosphatase conjugated goat anti-rabbit antibody (sc-2007, Santa Cruz Biotech for the AT1R and AT2R antibodies, and IgG 12-448, Upstate, for the MPO antibody) and CDP-Star substrate (Tropix, Bedford, MA, USA) were used to identify immunoreactive proteins by chemiluminescense. Images were captured by a cooled CCD camera (LAS-1000; Fujifilm, Tokyo, Japan) and semi quantification was done by comparing samples to positive controls using the Gauge 3.3 software (Fujifilm, Tokyo, Japan).

### Microscopic morphometry

Tissue specimens from the antral wall were fixed in Histofix (Histolab Products AB) and embedded in paraffin. Four-*μ *thick sections were mounted on coded glass slides and routinely stained with haematoxylin/eosin (H&E). The degree of mucosal infiltration of mononuclear and polymorphonuclear leucocytes was studied in the light microscope. The lamina propria will tend to increase in volume as a result of the influx of inflammatory cells. Therefore, the relative volume of the lamina propria was assessed by morphometry to reflect the infiltration of both mononuclear and polymorphonuclear leucocytes. This was carried out by H.F.H. in a light microscope, with a 10 × 10-square grid inserted into the eyepiece and a ×25 objective lens. Using the point counting method [22], the volume density of the lamina propria was determined and expressed in per cent of the mucosa (in this case defined as the region between the mucosal surface and the bottom of the glands). Mucosal volume is here defined as epithelial layer + lamina propria. The mucosal infiltration of polymorphonuclear leucocytes (PMNs) was assessed by P.H. in a light microscope, with a 10 × 10-square grid inserted into the eyepiece, a ×50 oil immersion objective lens (N.A. 1.4) and an oil immersion top lens of the condenser (N.A. 1.4). PMNs were identified as roughly round cells in lamina propria with a darkly stained multilobulated or bilobulated nucleus. The number of PMNs in a square region of the mucosa was determined, as was the total number of squares over the lamina propria; the number of PMNs is expressed per 1 mm^2 ^lamina propria. The counting started from the bottom of the glands and ended after assessment of one to seven full swaths of the mucosa, resulting in a minimum of 0.077 mm^2^lamina propria analyzed per section. Systematic sampling with a random start was used for selection of the areas to be studied in both analyses. Areas with lymphoid follicles were not included.

### Statistical analysis

The Kruskal-Wallis test and the Mann-Whitney U-test assessed significance in the differences of protein expression between the control, the TN2GF4-infected and the SS1-infected groups. The Mann-Whitney U-test assessed significance in differences in mucosal infiltration of inflammatory cells between the TN2GF4-infected and SS1-infected gerbils. A linear relationship between AT1R-protein and PMNs, and a linear relationship between AT1R and ln (MPO) in the antral mucosa, were tested with Pearson correlation. All tests were two-tailed, and statistical significance was set at p < 0.05. All the statistical analyses were carried out using SPSS, version 15.0 (SPSS Inc. Chicago, Illinois).

## Results

### Localization of AT1R and AT2R protein

Immunoreactivity to the AT1R and the AT2R proteins was observed in all gerbil specimens studied, and no staining was seen in the control sections. The AT1R antibody generally induced a more distinct staining than the AT2R antibody.

**Figure 1:**
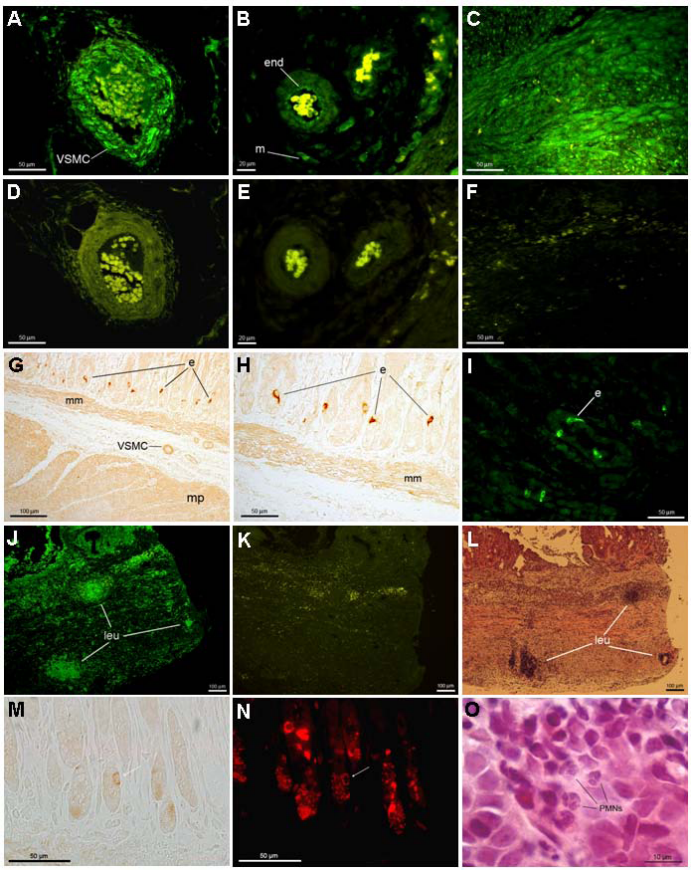
**Demonstrating the immunohistochemical location of AT1R and AT2R in the stomach of the Mongolian gerbil**. A-F shows sections from the antral region of an SS1-infected gerbil, 12 months after inoculation. **A**) Vascular smooth muscle cells (VSMC) in an artery located in the serosa stain positive for the AT1R protein (green color). **B**) Endothelial cells (end) and unidentified mesenchymal cells (m) located in the submucosa showing immunoreactivity for the AT2R protein. **C**) Smooth muscle cells in the muscular layer of the muscularis propria stain positive for the AT2R protein. **D**) A negative control (consecutive to the section in A) preabsorbed with the blocking peptide for the AT1R antibody, showing yellow autofluorescence from erythrocytes. **E & F**) Negative controls for the AT2R antibody, consecutive to the sections in B & C, respectively. **G, H **shows sections from the antral region of a control animal 6 months after inoculation and the section in (**I**) is from a TN2GF4-infected gerbil, 12 months after inoculation. Strong immunostaining for AT1R was found in cells with a typical appearance of endocrine cells (e). Muscularis mucosae (mm), muscularis propria (mp) and vascular smooth muscle cells (VSMC) also stained positive for the AT1R. A peroxidase based method (brown color) was used for staining of AT1R in G, H and immunofluorescence (green color) was used for staining of AT1R in (I). **J, K and L**) Consecutive sections from the antral region of a TN2GF4-infected gerbil, 6 months after inoculation. **J**) Immunostaining for AT1R (green color) shows aggregates of leucocytes (leu) expressing the AT1R protein. **K**) A negative control to the section in J, showing yellow autofluorescence from erythrocytes. **L**) The consecutive section to the section in K, routinely stained with H&E, confirming that the aggregates are leucocytes. **M and N **shows double immunostaining for AT1R (brown color in M) and for the pan-endocrine marker Chromogranin A (red color in N) of an antral section of a control animal. The white arrow in M and N points at a cell in the base of an antral gland that stained positive for both AT1R and Chromogranin A. Several of the Chromogranin A positive cells were not immunopositive to AT1R. **Section O **demonstrates neutrophils (PMNs) in the antral mucosa of an SS1-infected gerbil, 12 months after inoculation.

Several tissue compartments in both the corpus and antrum, independent of the presence or absence of *H. pylori *infection, exhibited immunoreactivity to both of the Ang II receptor subtypes (see Table 1 for an overview of the results and Figure 1 for representative sections. See also Additional file 1, 2, 3, 4, 5, 6, 7, 8, 9, 10, 11, 12, 13, 14 and 15 for high resolution images). Thus, immunoreactivity to both the AT1R and the AT2R were found in endothelial cells of vessels located in the lamina propria, the submucosa (Figure 1B) and the muscularis propria, as well as in smooth muscle cells of the muscularis mucosae and muscularis propria (Figure 1C and 1G) and in some mesenchymal cells located in the lamina propria and submucosa. Weak staining of both receptors was also noted, mainly in the basal part of most epithelial cells. Distinct staining of intramural neural structures was not observed. Immunoreactivity only to AT1R was observed in the vascular smooth muscle cells of vessels in the submucosa, the muscularis propria and the serosa of both the corpus and antral parts of the stomach (Figure 1A and 1G). Interestingly, a strong immunostaining for AT1R appeared in a subset of cells mainly in the base of the antral glands. These cells showed a typical endocrine cell appearance, i.e. they were quite small, with the cell bodies close to the basement membrane; a narrow string of cytoplasm was observed in some cases (Figure 1G, 1H and 1I). Such AT1R staining could not be found in endocrine-like cells in the oxyntic mucosa. Double immunostaining for AT1R and for the pan-endocrine marker Chromogranin A [23] confirmed the location of AT1R in a subpopulation of endocrine cells in the gerbil antral mucosa (Figure 1M and 1N).

**Table 1:** Localization of AT1R and AT2R protein in the gastric wall of the Mongolian gerbil

Tissue Compartment		Cell type	AT1R	AT2R
Mucosa	Epithelium	Most epithelial cells	+ mainly in basal parts	+ mainly in basal parts
		Small epithelial cells in the base of glands	+++ in some endocrine like cells in antrum	n.o.
	
	Lamina propria	Vasculature	+ in endothelium	+ in endothelium
		Nerve structures	n.o.	n.o.
		Mesenchymal cells	++ in some cells; ++ in leucocytes in *H. pylori*-infected	+ in some cells; ++ in leucocytes in *H. pylori*-infected
	
	Muscularis mucosae	Smooth muscle cells	++	+

Submucosa		Vasculature	+ in endothelium; ++ in VSMCs	+ in endothelium; n.o. in VSMCs
		Nerve structures	n.o.	n.o.
		Mesenchymal cells	++ in some cells; ++ in leucocytes in *H. pylori*-infected	+ in some cells; ++ in leucocytes in *H. pylori*-infected

Muscularis propria		Smooth muscle cells	++	+
		Vasculature	+ in endothelium; ++ in VSMCs	+ in endothelium; n.o. in VSMCs
		Nerve structures	n.o.	n.o.

Serosa		Vasculature	n.o. in endothelium; ++ in VSMCs	n.o.

n.o: not observed (or unspecific) immunoreactivity

+: weak immunoreactivity but with distinct location

++: easily recognised immunoreactivity

+++: strong immunoreactivity

VSMCs: vascular smooth muscle cells

The intensity and distribution of the immunoreactivity described above did not seem to be different between *H. pylori*-negative and *H. pylori*-infected animals or between the animals sacrificed at six or 12 months after inoculation. However, one obvious difference was noted between *H. pylori*-negative and *H. pylori*-infected animals: unlike in the uninfected gerbils, the sections of both the antrum and corpus of infected animals showed an abundance of inflammatory cells in the lamina propria with immunoreactivity to both the AT1R and the AT2R. In one of the TN2GF4-infected animals sacrificed at six months, aggregates of inflammatory cells were also seen in the muscularis propria (Figure 1J, 1K and 1L). No obvious differences in staining patterns or intensity were seen between animals infected with the TN2GF4 strain or the SS1 strain, or between animals sacrificed at six or 12 months after inoculation.

### Quantification of AT1R and AT2R protein and relation to inflammatory cells

Due to the similar immunohistochemical appearance of angiotensin II receptors in the corpus and antrum (with the exception of the AT1R expressing subpopulation of endocrine cells mentioned above) at both six and 12 months, quantitative assessments were restricted to antral specimens at 12 months after inoculation.

**Figure 2:**
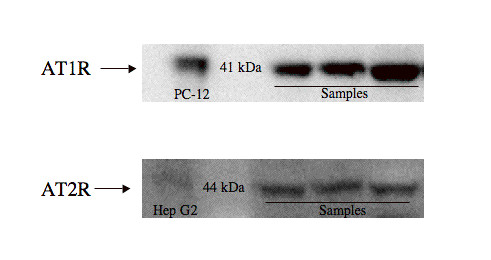
**Exemplary image of the western blots**. Top panel: Immunoreactivity against AT1R at 41 kDa in PC-12 cell lysate (positive control) and in antral full-wall samples. Bottom panel: Immunoreactivity against AT2R indicating a faint band at 44 kDa in Hep G2 (positive control) and bands in antral full-wall samples.

**Figure 3:**
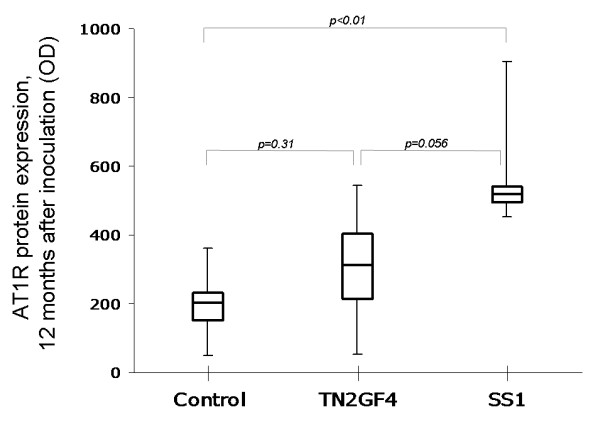
**Boxplot showing AT1R protein expression 12 months after inoculation in gerbils infected with *H. pylori *strains TN2GF4 and SS1 and in controls**. The AT1R protein expression was significantly higher in the SS1-infected animals compared with the controls (Kruskal-Wallis test and Mann-Whitney U test). Protein levels are presented as optical density (OD). Median values are indicated by the transverse line within the box, the interquartile range by the vertical extent of the box and the total range by the whiskers.

Both AT1R and AT2R proteins were identified by western blotting in all samples (Figure 2). After 12 months of infection, the AT1R protein expression was significantly higher in SS1-infected animals than in controls at that time (Figure 3). No significant differences in AT2R protein expression were found between the different groups of gerbils (not shown).

Given that *H. pylori*-infected animals showed an abundance of AT1R expressing inflammatory cells in their gastric mucosa and an increased antral AT1R expression, a quantitative analysis of the inflammatory cells in the mucosa was made to assess whether the up-regulation of AT1R protein in SS1-infected gerbils was related to mucosal infiltration of inflammatory cells.

The degree of mucosal infiltration of both mononuclear and polymorphonuclear leucocytes (reflected by the volume density of the lamina propria) did not differ between SS1-infected and TN2GF4-infected gerbils. However, the mucosal infiltration of polymorphonuclear leucocytes (PMNs) was significantly higher in the SS1-infected animals than in TN2GF4-infected animals 12 months after inoculation (Table 2). The PMNs in the *H. pylori*-infected mucosa consisted almost exclusively of neutrophils (Figure 1O); only a few eosinophilic and no basophilic cells could be identified in the microscope.

**Table 2:** Quantitative analysis of inflammatory cells in gerbil antral mucosa after 12 months of *Helicobacter pylori *infection

	Control	TN2GF4	SS1
Mucosal infiltration of mononuclear and polymorphonuclear leucocytes (reflected by the volume density (%) of lamina propria)	26.7 ± 2.4	47.1 ± 2.2	41.3 ± 2.4

Mucosal infiltration of polymorphonuclear leucocytes (PMNs/mm^2 ^lamina propria)	198 ± 37	1892 ± 169	4166 ± 275**

Values are mean ± standard error of mean.

**p < 0.01, SS1 versus TN2GF4. Mann-Whitney U-test

**Figure 4:**
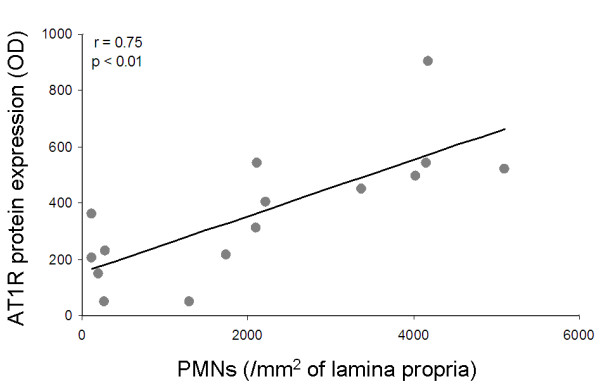
**Relationship between AT1R expression in the antrum and number of polymorphnuclear leucocytes (PMNs) in the antral mucosa after 12 months of *H. pylori *infection**.

**Figure 5:**
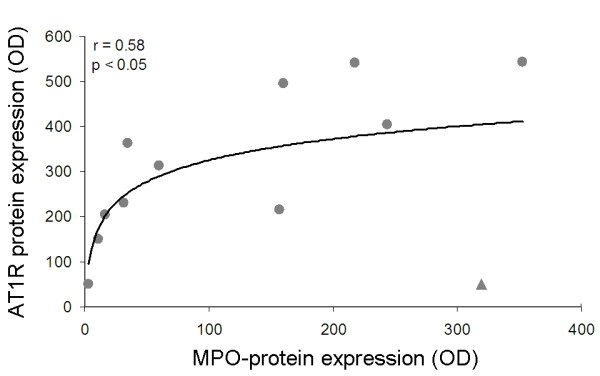
**Relationship between AT1R expression and myeloperoxidase (MPO) expression in the antral mucosa after 12 months of *H. pylori *infection**. If the outlier value marked with a triangle is excluded from the analysis (not shown) the relationship between AT1R and MPO becomes noticeably stronger (r = 0.86 and p < 0.01).

A linear relationship (r = 0.75, p < 0.01) was seen between the AT1R protein expression in the antrum and the number of polymorphonuclear leucocytes in the antral mucosa after 12 months of *H. pylori *infection (Figure 4). This relationship was supported by the logarithmic correlation between the antral expression of AT1R and the expression of myeloperoxidase (r = 0.58, p < 0.05) (Figure 5). Myeloperoxidase (MPO) is an enzyme that is abundantly expressed in neutrophils and to a lesser extent in monocytes and certain types of macrophages [24]. Hence, tissue levels of MPO served as protein markers of neutrophil infiltration in this study. If the outlier value marked with a triangle in Figure 5 is excluded from the analysis (not shown in a separate figure) the relationship between AT1R and MPO becomes noticeably stronger (r = 0.86 and p < 0.01).

## Discussion

The present study explored the baseline presence and location of angiotensin II receptors in the antral and corporal wall of the Mongolian gerbil and demonstrated that AT1R and AT2R were expressed by a variety of cells. A finding of particular interest was that a subpopulation of endocrine cells in the antral mucosa showed a marked expression of AT1R. The validity of the immunohistochemical procedure used in the present study was confirmed in various ways. The Ang II receptor antibodies were detected using two different techniques, and the specificity of the primary antibodies used for both western blotting and immunohistochemistry was checked by comparing the staining patterns of the gerbil with those of the human adrenal gland and by preabsorption with blocking peptide (AT1R).

The ubiquitous distribution of Ang II receptors in the stomach reported here is in concordance with what has been reported earlier in the colon [7]. Autoradiography of the rat stomach has also indicated that AT1R, and low numbers of AT2R, are present in all layers of the stomach [4]. However, the precise location of AT1R and AT2R in the stomach has as yet only been sparsely explored. Matsuo *et al*. used immunohistochemistry to locate AT1R in human antrum and found it in vascular smooth muscle cells (VSMCs), mesenchymal cells and smooth muscle cells in the muscular layers of the mucosa and muscularis propria [25]. Further, Bregonzio *et al*. located the AT1R protein by means of immunohistochemistry in vascular endothelial cells in the rat stomach [4].

Previous studies have reported actions of Ang II on gastric smooth muscle contraction and gastric mucosal blood flow [2,3]. Thus, the Ang II receptors found in this study in gastric smooth muscle cells in the muscularis propria and VSMCs of arteries in the submucosa most likely mediate these effects, respectively. We found both AT1R and AT2R on endothelial cells of vessels in all layers of the stomach, and it has previously been suggested that AT1R stimulation of vascular endothelial can increase gastric microvascular permeability [11]. Several studies indicate that activation of the AT2R has effects that oppose those mediated by the AT1R, thus modulating the responses to stimulation with Ang II. Furthermore, in the present study, a weak but distinct immunoreactivity for AT2R and AT1R was seen primarily in the basal part of most epithelial cells. The physiological function of these receptors is unclear but could speculatively involve sodium, water or alkaline secretions, or regulation of cell growth [8,26].

The location of AT1R in a subpopulation of endocrine cells in the antral mucosa was an unexpected finding that to the best of our knowledge has not been reported previously. An AT1R mediated influence on hormonal release from gastric endocrine cells could theoretically provide the basis for a previously unknown regulatory link between RAS and gastric functions. It has been reported that Ang II through AT1R can influence gastric acid secretion in Na+ depleted rats [1]. It can be speculated that this influence can involve stimulation or inhibition of hormonal release from gastric endocrine cells containing gastrin, somatostatin or histamine. Interestingly, Wong *et al*. showed that somatostatin producing cells in the rat pancreas express the AT2R protein, and that activation of the AT2R inhibits Ang II stimulated somatostatin release in rat pancreatic cell lines [27]. The location of AT1R in endocrine antral cells may have interesting physiological and pathophysiological consequences, and future studies are needed to evaluate this finding.

The immunohistochemical analysis in this study demonstrated that Ang II receptors were located in both the plasma membrane and in the cytosol. The cytosolic presence of the membrane bound Ang II receptors may seem odd but can be regarded as a state of internalization of the receptors. Rattan and de Godoy reported that Ang II concentration dependent translocation of the Ang II receptors occurs between the plasma membrane and the cytosol in smooth muscle cells of the rat internal anal sphincter [28]. Moreover, recent studies have also provided evidence of a complete, functional RAS within cells, described as an intracellular RAS system [29].

The present study also investigated whether *H. pylori *infection influences the expression of the gastric Ang II receptors and demonstrated that AT1R and AT2R were expressed by inflammatory cells infiltrating the *H. pylori*-infected stomach and that infection with *H. pylori *strain SS1 significantly increased antral AT1R protein expression and the number of PMNs in the antral mucosa.

As previously reported by Elfvin *et al*., the SS1-infected gerbils showed hardly any pathologic lesions at 12 months [14]. This was in contrast with infection with strain TN2GF4, which caused gastric ulcers, gastric intestinal metaplasia and gastric cancer-like lesions [14]. When this is taken together with the present results, it can be speculated that the TN2GF4 strain, known to be very virulent, may contribute to lesional injuries by suppression of AT1R expression and infiltration by PMNs.

The correlations found in this study between AT1R expression and the number of antral PMNs and between AT1R and MPO expression in the antral mucosa can be explained either by higher AT1R expression in PMNs than in mononuclear leucocytes or by mucosal AT1R expression that increases the infiltration of PMNs. Because immunohistochemistry is a suboptimal method for quantifying protein expression, we cannot conclude from our observations in the present investigation whether or not the PMNs had a higher AT1R expression than the mononuclear leucocytes. Rasini *et al*. offer support for higher AT1R expression in PMNs than in mononuclear leucocytes. Using flow cytometry and RT-PCR, these authors showed that PMNs in human venous blood have a higher AT1R expression than the other leucocytes [30]. It is also known that AT1R stimulation of leucocytes can generate reactive oxygen species such as hydrogen peroxide (H_2_O_2_), and we have recently shown that SS1-infected gerbils have a higher juxtamucosal H_2_O_2 _production than TN2GF4-infected gerbils [31]. In addition, Nakagiri *et al*. report that blocking AT1R decreases gastric mucosal H_2_O_2 _production in rats [5]. Theoretically, it could be beneficial for the TN2GF4 bacteria to inhibit PMNs' AT1R expression to keep H_2_O_2 _production at a level that is optimal for its survival.

On the other hand, support for the possibility that *H. pylori *infection induces mucosal AT1R expression that increases the infiltration of PMNs is that AT1R antagonists decrease neutrophil infiltration in a rat model of stress induced gastric injury, as well as in a rat model of indomethacin induced enteritis [4,32]. We found AT1R on the endothelial cells of vessels located in the gerbil mucosa, and Matsuo *et al*. have previously shown that *H. pylori *infection increases the human gastric mucosal mast cell chymase expression [25]. Mast cell chymase contributes to Ang II formation, and this Ang II can hypothetically stimulate AT1R expressed on endothelial cells or other cells in the *H. pylori*-infected gastric mucosa, attracting PMN infiltration by the induction of endothelial adhesion proteins such as ICAM-1 [4].

Future experiments using RAS blockers are required to evaluate the causality of the relationship between AT1R expression and PMN infiltration found in this study.

A causal relationship between AT1R and PMN infiltration is interesting since it may constitute a basis for the recently reported epidemiological associations between RAS related gene polymorphism and the development of sequelae to *H pylori *infection [12,13].

The results indicate that, unlike the situation in stress induced or ischemia/reperfusion induced gastric injury [4,5], the AT1R may have beneficial effects to the host in *H pylori*-induced gastritis. AT1R antagonists are commonly prescribed to humans as antihypertensives. Consequences for patients with *H. pylori *associated gastritis are unknown and represent an interesting field for future research.

## Conclusions

The present study demonstrates angiotensin II receptors in the antral and corporal wall of the Mongolian gerbil. AT1R and AT2R are expressed by a variety of cells, including a subpopulation of endocrine cells in the antral mucosa and infiltrating leucocytes in the *H. pylori*-infected stomach. The results indicate an influence on the gastric AT1R expression that is dependent on the *H. pylori *strain as well as a relationship between gastric AT1R expression and mucosal PMN infiltration.

## Competing interests

The authors declare that they have no competing interests.

## Authors' contributions

P.H. participated in the coordination of the study, the immunoassays and the microscopic morphometry and carried out the statistical analyses and drafted the manuscript. A.C. participated in the immunoassays and in the writing of the manuscript. H.F.H. participated in the microscopic morphometry, the interpretation of immunohistochemistry and the writing of the manuscript. A.E. and L.F. participated in the conception of the study and its design and coordination and in the writing of the manuscript. All authors read and approved the final manuscript.

## Pre-publication history

The pre-publication history for this paper can be accessed here:

http://www.biomedcentral.com/1471-230X/10/3/prepub

## Supplementary Material

Additional file 1 **Figure 1A**. High resolution image (TIF) of Figure 1A Click here for file

Additional file 2 **Figure 1B**. High resolution image (TIF) of Figure 1B Click here for file

Additional file 3 **Figure 1C**. High resolution image (TIF) of Figure 1C Click here for file

Additional file 4 **Figure 1D**. High resolution image (TIF) of Figure 1D Click here for file

Additional file 5 **Figure 1E**. High resolution image (TIF) of Figure 1E Click here for file

Additional file 6 **Figure 1F**. High resolution image (TIF) of Figure 1F Click here for file

Additional file 7 **Figure 1G**. High resolution image (TIF) of Figure 1G Click here for file

Additional file 8 **Figure 1H**. High resolution image (TIF) of Figure 1H Click here for file

Additional file 9 **Figure 1I**. High resolution image (TIF) of Figure 1I Click here for file

Additional file 10 **Figure 1J**. High resolution image (TIF) of Figure 1J Click here for file

Additional file 11 **Figure 1K**. High resolution image (TIF) of Figure 1K Click here for file

Additional file 12 **Figure 1L**. High resolution image (TIF) of Figure 1L Click here for file

Additional file 13 **Figure 1M**. High resolution image (TIF) of Figure 1M Click here for file

Additional file 14 **Figure 1N**. High resolution image (TIF) of Figure 1N Click here for file

Additional file 15 **Figure 1O**. High resolution image (TIF) of Figure 1O Click here for file
