# Supplementary figures and images for: Angiotensin II receptor expression and relation to Helicobacter pylori-infection in the stomach of the Mongolian gerbil
Source: BMC Gastroenterol. 2010 Jan 14;10:3. doi: 10.1186/1471-230X-10-3 (PMC2823647; doi:10.1186/1471-230X-10-3)

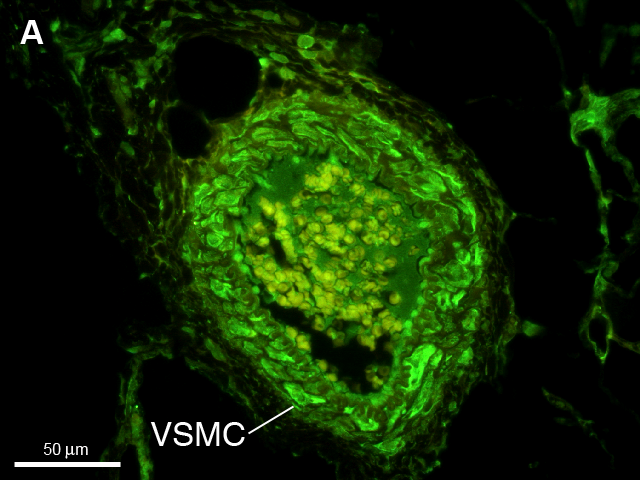

Supplement: Additional file 1 — Figure 1A. High resolution image (TIF) of Figure 1A [file 1471-230X-10-3-S1.TIFF]

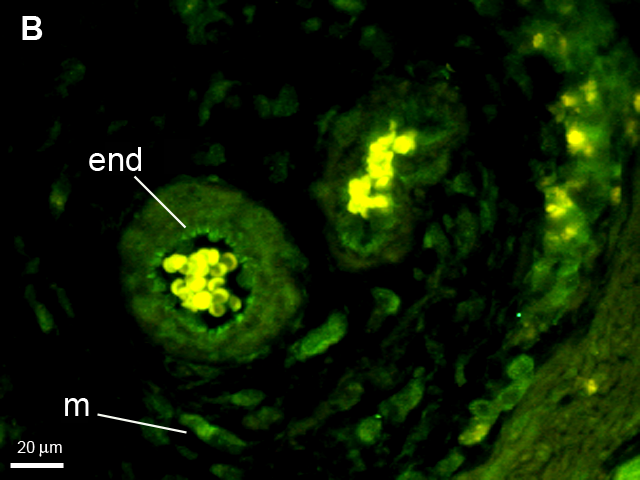

Supplement: Additional file 2 — Figure 1B. High resolution image (TIF) of Figure 1B [file 1471-230X-10-3-S2.TIFF]

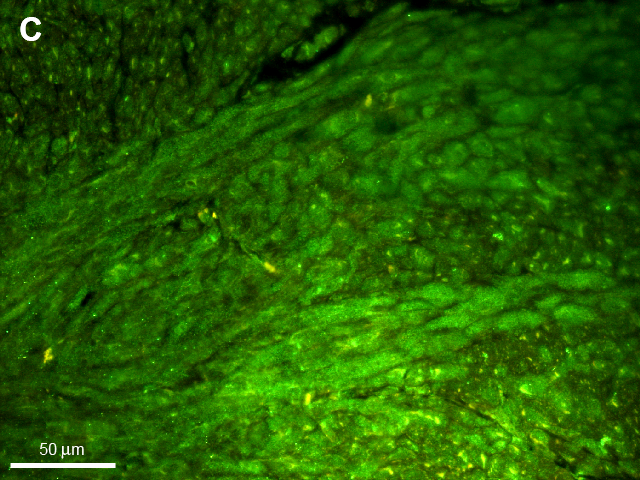

Supplement: Additional file 3 — Figure 1C. High resolution image (TIF) of Figure 1C [file 1471-230X-10-3-S3.TIFF]

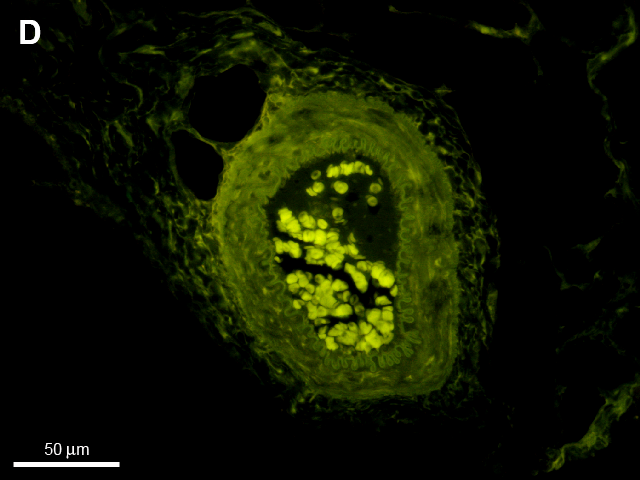

Supplement: Additional file 4 — Figure 1D. High resolution image (TIF) of Figure 1D [file 1471-230X-10-3-S4.TIFF]

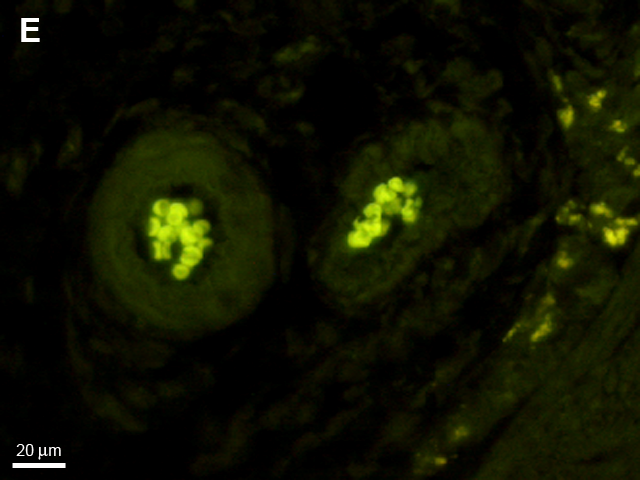

Supplement: Additional file 5 — Figure 1E. High resolution image (TIF) of Figure 1E [file 1471-230X-10-3-S5.TIFF]

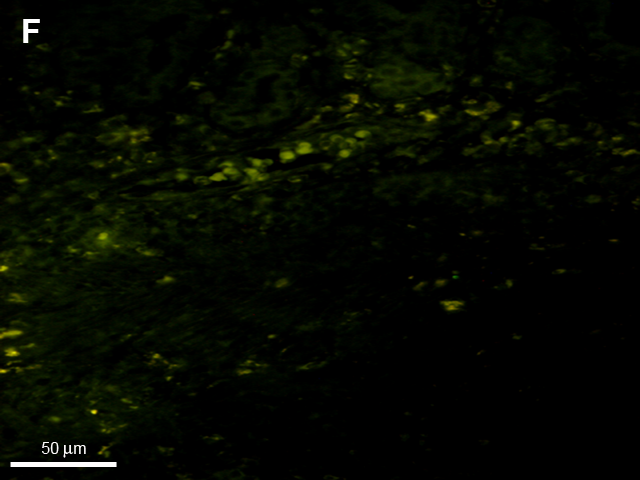

Supplement: Additional file 6 — Figure 1F. High resolution image (TIF) of Figure 1F [file 1471-230X-10-3-S6.TIFF]

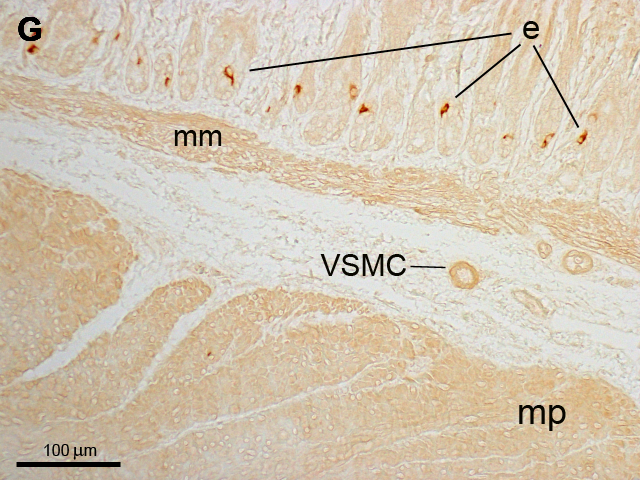

Supplement: Additional file 7 — Figure 1G. High resolution image (TIF) of Figure 1G [file 1471-230X-10-3-S7.TIFF]

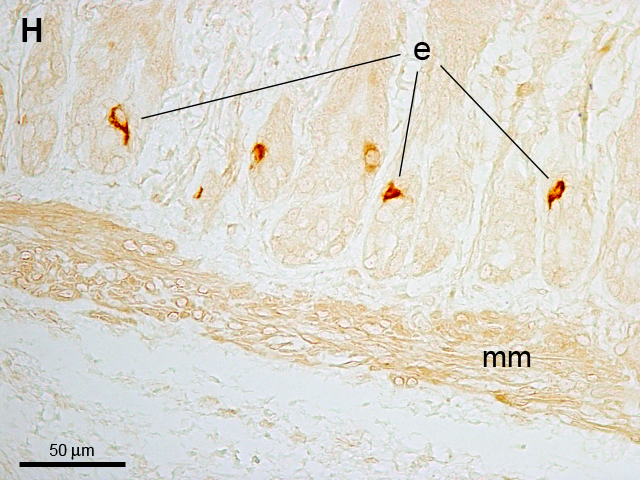

Supplement: Additional file 8 — Figure 1H. High resolution image (TIF) of Figure 1H [file 1471-230X-10-3-S8.TIFF]

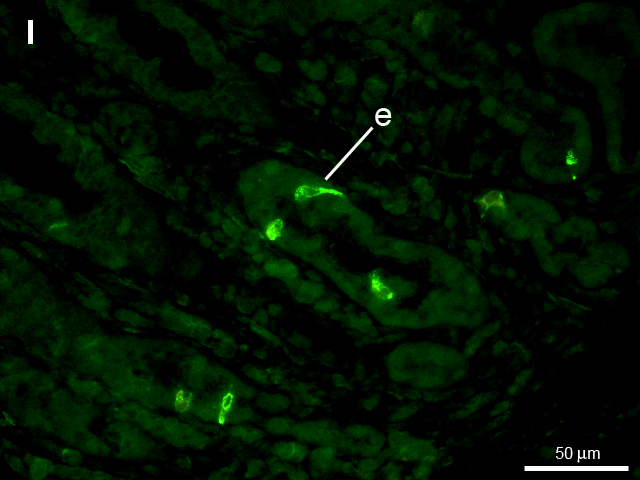

Supplement: Additional file 9 — Figure 1I. High resolution image (TIF) of Figure 1I [file 1471-230X-10-3-S9.TIFF]

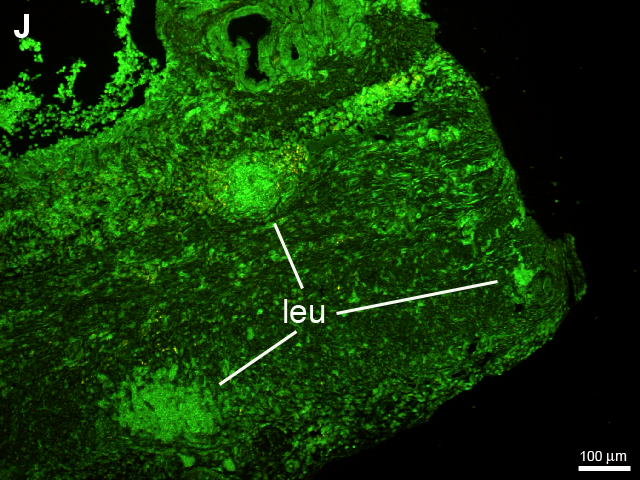

Supplement: Additional file 10 — Figure 1J. High resolution image (TIF) of Figure 1J [file 1471-230X-10-3-S10.TIFF]

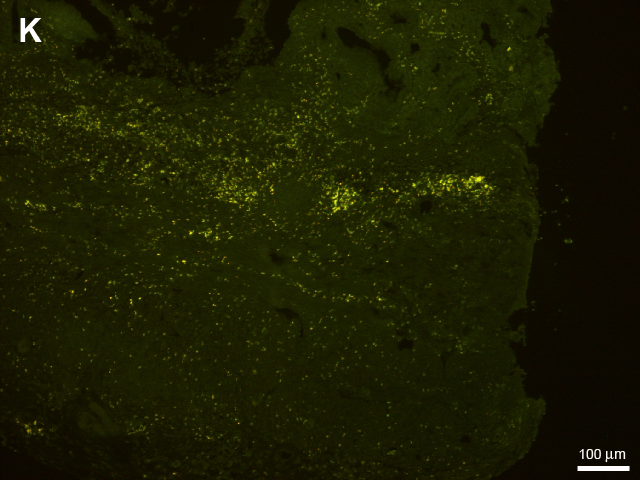

Supplement: Additional file 11 — Figure 1K. High resolution image (TIF) of Figure 1K [file 1471-230X-10-3-S11.TIFF]

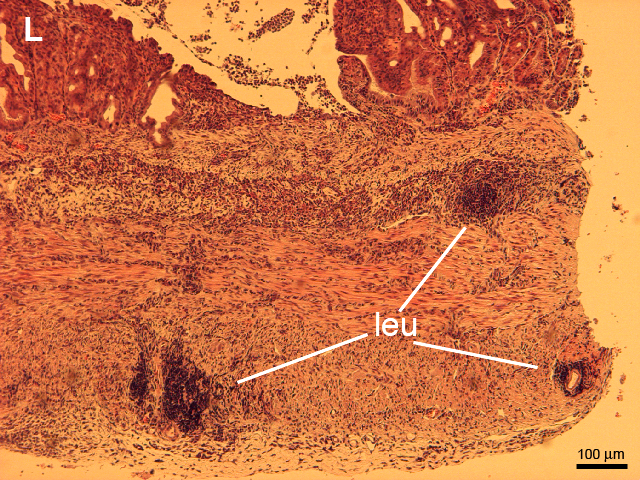

Supplement: Additional file 12 — Figure 1L. High resolution image (TIF) of Figure 1L [file 1471-230X-10-3-S12.TIFF]

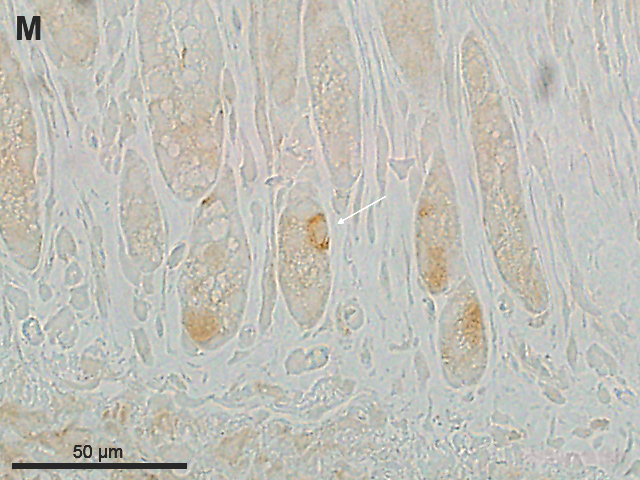

Supplement: Additional file 13 — Figure 1M. High resolution image (TIF) of Figure 1M [file 1471-230X-10-3-S13.TIFF]

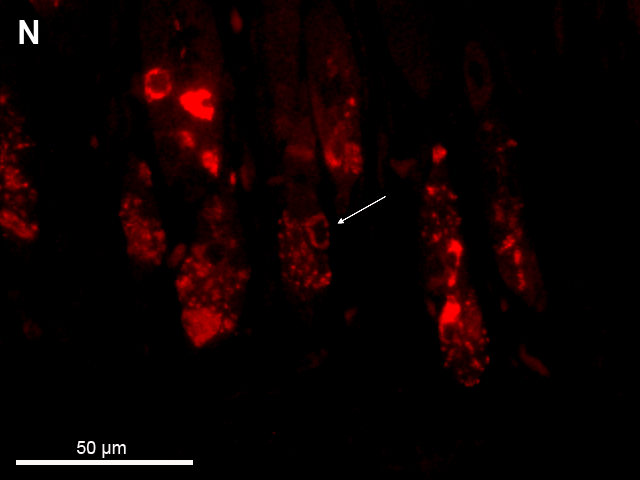

Supplement: Additional file 14 — Figure 1N. High resolution image (TIF) of Figure 1N [file 1471-230X-10-3-S14.TIFF]

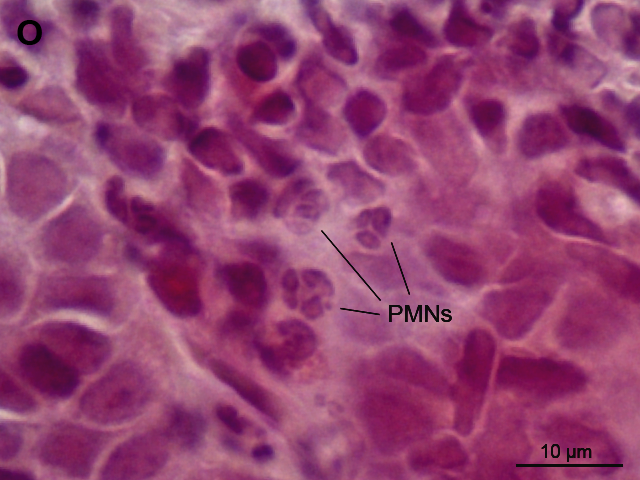

Supplement: Additional file 15 — Figure 1O. High resolution image (TIF) of Figure 1O [file 1471-230X-10-3-S15.TIFF]
